# Supplementary material for: The Systems Biology Research Tool: evolvable open-source software
Source: BMC Syst Biol. 2008 Jun 29;2:55. doi: 10.1186/1752-0509-2-55 (PMC2446383; doi:10.1186/1752-0509-2-55)
Supplement: Additional file 1 — SBRT Archive. An archive of the current version of the Systems Biology Research Tool. [file 1752-0509-2-55-S1.zip › sbrt-1.4.0/doc/developers_guide/api/sbrt/shell/mng/fba/package-frame.html]

sbrt.shell.mng.fba


sbrt.shell.mng.fba

Classes 
  
BiggSbmlReaderManager
  
BiggSbmlTranslaterManager
  
CatalystDeletionManager
  
CdHarFluxVectorManager
  
CvCatalystDeletionManager
  
CvFbaOptManager
  
CvMsFluxIntervalManager
  
CvObjectiveFunctionManager
  
CvRevRxnFluxIntervalManager
  
CvRxnDeletionManager
  
CvSimpleFluxIntervalManager
  
ExtremeCurrentManager
  
ExtremeCurrentManager.Rm
  
FbaManager
  
FbaOptManager
  
FbaSolverManager
  
FluxActivityManager
  
FluxCapManager
  
FluxPlasticityManager
  
InitialPointManager
  
IntervalVariationManager
  
MetatoolFileWriterManager
  
MmaNotebookManager
  
MsFluxIntervalManager
  
MsFluxomeReducerManager
  
NetworkInfoManager
  
ObjectiveFunctionManager
  
PalssonSbmlReaderManager
  
PalssonSbmlTranslaterManager
  
RandomConstraintsManager
  
RandomEnvManager
  
RandomFluxVectorManager
  
RandomObjectiveManager
  
ReverseRxnFluxIntervalManager
  
RxnDeletionManager
  
RxnRemovalManager
  
SbmlWriterManager
  
SimpleFluxIntervalManager
  
SimpleRxnFileTransManager
  
SlpCycleIdManager
  
StoichVariationManager
  
SvCdHarFluxVectorManager
  
SvInitialPointManager
  
WwFluxomeReducerManager
